# Inflammatory cardiomyopathies: short- and long-term outcomes after heart transplantation—a protocol for a systematic review and meta-analysis

**DOI:** 10.1007/s10741-020-09919-x

**Published:** 2020-01-14

**Authors:** Emanuele Bobbio, Marie Lingbrant, Bright I Nwaru, Eva Hessman, Jukka Lehtonen, Kristjan Karason, Entela Bollano

**Affiliations:** 1 Department of Cardiology, Sahlgrenska University Hospital, Gothenburg, Sweden; 2 Krefting Research Centre, Institute of Medicine, University of Gothenburg, Gothenburg, Sweden; 3 University Library, University of Gothenburg, Gothenburg, Sweden; 4 Heart and Lung Center, Helsinki University and Helsinki University Hospital, Helsinki, Finland; 5 Department of Cardiothoracic Surgery, Sahlgrenska University Hospital, Gothenburg, Sweden; 6 Transplant Institute, Sahlgrenska University Hospital, Gothenburg, Sweden; 7 Institute of Medicine at Sahlgrenska Academy, University of Gothenburg, Gothenburg, Sweden

**Keywords:** Inflammatory cardiomyopathies (ICM), Cardiac sarcoidosis (CS), Giant cell myocarditis (GCM), Heart transplantation, Meta-analysis, Protocol and guidelines, Systematic review

## Abstract

Heart transplantation (HTx) for patients with “giant cell myocarditis” (GCM) or “cardiac sarcoidosis” (CS) is still controversial. However, no single center has accumulated enough experience to investigate post-HTx outcome. The primary aim of this systematic review is to identify, appraise, and synthesize existing literature investigating whether patients who have undergone HTx because of GCM or CS have worse outcomes as compared with patients transplanted because of other etiologies. A systematic and comprehensive search will be performed using PubMed, Scopus, Web of Science, EMBASE, and Google Scholar, for studies published up to December 2019. Observational and interventional population-based studies will be eligible for inclusion. The quality of observational studies will be assessed using the Newcastle–Ottawa scale, while the interventional studies will be assessed using the Cochrane Effective Practice Organization of Care tool. The collected evidence will be narratively synthesized; in addition, we will perform a meta-analysis to pool estimates from studies considered to be homogenous. Reporting of the systematic review and meta-analysis will be in accordance with the Meta-analysis of Observational Studies in Epidemiology Preferred Reporting Items for Systematic reviews and Meta-Analysis guidelines. To our knowledge, this will be the first synthesis of outcomes, including survival, acute cellular rejection, and disease recurrence, in patients with either GCM or CS treated with HTx. Reviewing the suitability of HTx in this population and highlighting areas for further research will benefit both patients and healthcare providers. Trial registration: CRD42019140574.

## Introduction

Inflammatory cardiomyopathies (ICMs) refer to a broad group of disorders in which myocardial inflammation is the primary cause of cardiac dysfunction. The natural history is highly variable and clinical features vary from slight symptoms to life-threatening heart failure (HF) and arrhythmias [1]. Infectious etiologies of ICMs are the most studied, with viral myocarditis being the best understood. However, it is imperative to consider other causes of inflammation in order to ensure that the patient receives the appropriate diagnostic and therapeutic possibilities. Among them, there is a variety of autoimmune conditions that can involve chronic inflammation, as either a primary or a secondary feature, affecting the heart by diverse mechanisms [1].

Sarcoidosis is a multisystem disease characterized by non-caseating granulomatous inflammation that can display a wide array of manifestations in different organ systems and show various degrees of severity. Most patients present with pulmonary involvement but in practice, any organ can be affected [1]. Based on autopsy studies, up to one-third of patients with sarcoidosis have cardiac involvement, although in less than 10%, this is clinically apparent [2–4]. The diagnosis of cardiac sarcoidosis (CS) has increased dramatically over the past 25 years, with a 20-fold increase in the detection rate of CS being observed [5]. This increase is likely due to enhanced awareness of the condition, improved imaging techniques, and development of standardized diagnostic algorithms. In the case of clinically isolated cardiac involvement, the diagnosis can be extremely challenging and examination of the explanted heart can be the only way to identify myocardial sarcoid granulomas [5, 6]. Investigation of outcomes in this population has been limited to small case series and reports due to disease rarity and difficulties with diagnosis and registry classification [7].

Heart transplantation (HTx) is a fairly well-established treatment for patients with advanced HF due to CS [6, 8]. However, it has been performed in a relatively small number of cases, partly because of the rarity of the disease, and partly because HTx is contraindicated in cases of extensive and uncontrolled extra-cardiac involvement. Moreover, there have been concerns about long-term morbidity in this population due to the risk of recurrence of CS in the transplanted heart, development of secondary organ failure due to systemic sarcoidosis, and progression of deleterious effects of pulmonary sarcoidosis on right ventricular and pulmonary hemodynamics [5, 9, 10].

CS recurrence post-transplant has been reported, but appears to be rare [11–13]. One of the limitations of assessing recurrence of CS is the poor sensitivity of biopsies in detecting CS, given the patchy nature of the disease. According to single-center follow-up studies, the rates of rejection and overall mortality appear to be similar in recipients with CS as compared with those who have undergone HTx for other causes [6, 14–16].

Giant cell myocarditis (GCM) is a rare inflammatory heart disease commonly depicted as rapidly progressive and usually fatal and for which HTx is the treatment of choice [17]. The disease caught attention in the 1990s when the International Multicenter GCM Study Group described that 89% of the 63 included patients with GCM either underwent HTx or died, with the median survival being only 5.5 months [17]. In a later analysis, transplant-free survival at 5 years in patients with GCM was reported to be as low as 10% overall and in patients diagnosed by endomyocardial biopsy (EMB) 22% [18]. Since most of the patients were diagnosed after HTx or at autopsy, these data may not reflect the actual situation. Although more recent studies have reported improved survival, the outcomes of patients with GCM who have undergone HTx are still poorly investigated [19–22]. Recurrence of GCM in the cardiac graft has been reported as high as 20–25%, and early studies suggested an increased risk of early rejection in this patient population [17–21, 23]. However, there are no contemporary data describing the short- and long-term outcomes of GCM after HTx.

No single center has accumulated enough experience to investigate outcomes in patients with either GCM or CS and debate exists regarding the suitability of HTx due to the possibility of disease recurrence and involvement of other organs. Therefore, to gain clearer insights into this field, it is paramount to identify and synthesize the available evidence to date.

## Study aim and objectives

The primary aim of this systematic review is to identify, critically appraise, and synthesize existing literature investigating whether patients who have undergone HTx because of GCM or CS have worse rate of survival compared with patients transplanted for other heart failure etiologies. A secondary aim of the review is to compare rates of acute cellular rejection and disease recurrence between (1) the GCM and non-GCM groups and (2) the CS and non-CS groups.

## Methods and analysis

The protocol for this review was developed in accordance with the recommendation of the preferred reporting items for systematic review and meta-analysis protocols (PRISMA-P) statement and has been registered in PROSPERO (registration number: CRD42019140574) [24]. The systematic review will be performed according to the Meta-analysis of Observational Studies in Epidemiology (MOOSE) guidelines and will be reported according to the Preferred Reporting Items for Systematic reviews and Meta-Analysis (PRISMA) guidelines [25, 26].

### Ethical issues

This systematic review does not require ethical approval or informed consent because it will be based only on previously published data and does not implicate any direct contact with individual patients.

### Eligibility criteria

The population of interest will be patients that have been treated with HTx due to either GCM or CS. We will include studies that report rates of survival, rates of acute cellular rejection and disease recurrence, or the number of subjects who achieved the outcomes (survival, acute cellular rejection, and disease recurrence), from which the incidence can be calculated. Data across different age groups, gender, and ethnicity will be analyzed, and there will be no exclusion by country or language. We will translate studies reported in other languages than English.

We will include observational (cross-sectional, case–control, cohort) and interventional (randomized controlled trials, community trials, field trials) population-based studies. Review articles, non-peer reviewed articles, commentaries, proceedings, laboratory science studies, case studies, case series, and other studies that do not allow the calculation of rates of the outcomes will be excluded from this study.

If an eligible study does not report the incidence of the pre-specified outcomes or does not report any, we will attempt to contact the study authors to request for the respective details.

### Information sources

Our sources of information will include electronic databases, different types of gray literature, conference abstracts, trial registries, and researchers and authors themselves. An electronic search will be performed through PubMed, Scopus, Web of Science, EMBASE, and Google Scholar employing popular and commonly used phrases stated in related literature, in addition to Medical Subject Headings (MeSH) terms. All databases will be searched from their inception date until the end of December 2019. Reference lists of included studies will be examined for additional relevant articles. Articles retrieved from the databases will be exported to Endnote for removal of duplicates and further screening.

### Search strategy

Our initial search syntax for PubMed is given below and this will be adapted in searching the other databases.

1. “giant cell”[tiab] OR “giant cell”[mesh]
2. Myocarditis[tiab] OR myocarditis[mesh]
3. (“giant cell”[tiab] OR “giant cell”[mesh]) AND (Myocarditis[tiab] OR myocarditis[mesh])
4. (Sarcoidosis[tiab] OR sarcoidosis[mesh] OR sarcoid[tiab]) AND (cardiac[tiab] OR heart[tiab] OR heart[mesh])
5. Inflammatory [tiab] AND (cardiomyopathy[tiab] OR cardiomyopathies[tiab])
6. (“cardiac transplant”[tiab] OR “cardiac transplant”[mesh] OR “heart transplantation”[mesh] OR “heart transplant”[tiab])
7. #3 AND #6
8. #4 AND #6
9. #5 AND #6
10. #7 OR #8 OR #9

### Study records

#### Selection process

Two reviewers will independently perform the primary article screening. Firstly, they will review the titles and abstracts of the articles, and, secondly, categorize the selected articles into three groups: relevant, irrelevant, and unsure. Articles categorized as irrelevant by both reviewers will be eliminated from the review. For articles determined to be eligible (relevant or unsure articles) based on the title or abstract, the full papers will be retrieved. Potentially relevant articles chosen by at least one author will be retrieved and the full text evaluated. Then, the full text of the eligible articles will be reviewed and a list of articles to be included will be made by each reviewer. The two lists will then be compared and non-conformities will be discussed. The whole team will make the final decision when an agreement is not reached. A flow diagram for the search and selection process will be developed using the PRISMA guidelines.

#### Data extraction

A data extraction form will be developed to extract relevant study data from all included studies. This will help standardize the information recorded and aid analyses. The following data will be extracted independently by two authors: study name (along with the name of the first author and year of publication), country where the study was conducted, source from which patients or study participants were selected, study design, outcomes definition, age, gender, ethnicity, incidence or prevalence with 95% confidence intervals (CIs) or the number of patients achieving the outcomes, and the total number of subjects. If the required data are inadequate, unclear, or missing from the article, authors will be contacted. If needed, missing information will be calculated from the available data when possible. The data extraction will be discussed by the two reviewers, and a third reviewer will arbitrate in the case of any disagreement.

### Quality assessment

The quality of the studies will be independently assessed by two authors using the Newcastle–Ottawa quality assessment scale (NOS), while the interventional studies will be assessed using the Cochrane Effective Practice Organization of Care tool. Critical appraisal of articles will be performed to assess the methodological quality of each article. The quality appraisal will be undertaken by two reviewers, and a third reviewer will arbitrate in the case of any disagreement.

### Data synthesis

We will calculate rate of survival and rates of acute cellular rejection and disease recurrence in each study if possible. Following this, and if the included studies are sufficiently homogeneous, we will undertake a meta-analysis by pooling estimates across studies using the random effects approach. Heterogeneity between studies will be assessed using the Higgins *I*^2^ statistic. *I*^2^ values of 0–30% will be considered as showing minimal heterogeneity, 31–50% moderate heterogeneity, and > 50% substantial heterogeneity [27]. We will assess the quality of the evidence from interventional studies using the Grading of Recommendations Assessment, Development, and Evaluation (GRADE) framework [28].

Begg’s funnel plot and Egger’s test will be used to assess publication bias. The former is a scatter plot of the log odds ratios (ORs) of individual studies on the *x*-axis against 1/standard error (SE) of each study on the *y*-axis. Egger’s test is a linear regression test of the normalized effect estimate (log OR/SE) against its precision (1/SE) [29]. *P* value of < 0.10 on Egger’s test or an asymmetrical funnel plot will be considered to indicate the presence of publication bias.

If data are sufficient, we will conduct subgroup analysis based on the quality of study, country, age, gender, and ethnicity.

## Discussion

Inflammatory cardiomyopathy may lead to advanced heart failure with poor prognosis to the extent that heart transplantation is required. The two inflammatory diseases that most frequently lead to HTx include cardiac sarcoidosis and giant cell myocarditis. Outcome after HTx in these patient cohorts have been described in single-center studies and appear to be acceptable. However, there is an ongoing debate regarding the suitability of HTx in patients with either GCM or CS due to the risk of disease recurrence and other factors that may contribute to an adverse post-HTx outcome.

This protocol presents the methodology of a systematic review for investigating short- and long-term outcomes in patients with either GCM or CS who have been treated with HTx. In addition, this review will study the influence of age, gender, ethnicity/race, and pharmacological treatment on the rates of survival, acute cellular rejection, and disease recurrence.

Although the topic has been studied previously, no single center has accumulated enough experience to draw definitive conclusions. Therefore, all reviews performed on this subject are at risk of different types of heterogeneity due to various populations, research designs, and study settings. Overall, we believe that our systemic review will provide clearer insights into this challenging and clinically important topic.
